# A De Novo Designed Esterase with p-Nitrophenyl Acetate Hydrolysis Activity

**DOI:** 10.3390/molecules25204658

**Published:** 2020-10-13

**Authors:** Guanlin Li, Li Xu, Houjin Zhang, Junjun Liu, Jinyong Yan, Yunjun Yan

**Affiliations:** 1 Key Laboratory of Molecular Biophysics, Ministry of Education, College of Life Science and Technology, Huazhong University of Science and Technology, Wuhan 430074, China; D201077390@alumni.hust.edu.cn (G.L.); xuli@mail.hust.edu.cn (L.X.); hjzhang@hust.edu.cn (H.Z.); yjiny@hust.edu.cn (J.Y.); 2 Wuhan Hiteck Biological Pharma Co., Ltd., Wuhan 430056, China; 3 School of Pharmacy, Tongji Medical College, Huazhong University of Science and Technology, 13 Hangkong Road, Wuhan 430030, China; junjun.liu@hust.edu.cn

**Keywords:** esterase, de novo design, hydrolysis activity

## Abstract

Esterases are a large family of enzymes with wide applications in the industry. However, all esterases originated from natural sources, limiting their use in harsh environments or newly- emerged reactions. In this study, we designed a new esterase to develop a new protocol to satisfy the needs for better biocatalysts. The ideal spatial conformation of the serine catalytic triad and the oxygen anion hole at the substrate-binding site was constructed by quantum mechanical calculation. The catalytic triad and oxygen anion holes were then embedded in the protein scaffold using the new enzyme protocol in Rosetta 3. The design results were subsequently evaluated, and optimized designs were used for expression and purification. The designed esterase had significant lytic activities towards p-nitrophenyl acetate, which was confirmed by point mutations. Thus, this study developed a new protocol to obtain novel enzymes that may be useful in unforgiving environments or novel reactions.

## 1. Introduction

Esterases are commonly used in the food, pharmaceutical, agricultural and chemical industries. The methods for obtaining new esterases mainly include mining existing natural biological resources such as extreme environment organisms, isolated bacterial genomes, and uncultured metagenomes [1,2]. However, esterases in nature are usually not suitable for the application in harsh environments or newly-emerged reactions.

Esterases account for a small portion of the protein sequence space [3]. Because evolution is driven by progressive mutations and selection pressure, the sequences of the native proteins are not evenly distributed throughout the sequence space. Instead, they form a family of proteins with similar amino acid sequences, structures, and functions [4]. Exploring the large sequence space outside the evolutionary path requires the de novo design of proteins [5,6,7]. To this end, a new protocol is needed to facilitate the design of novel enzymes.

The origin of de novo protein design can be traced back to pioneering work by DeGrado and coworkers who designed peptide inhibitors of calmodulin [8]. With the improvement of protein design algorithms and a better understanding of protein structures, this field has seen rapid development in the last two decades. Since the turn of the century, many milestones have been accomplished by David Baker’s lab. In 2003, Kuhlman, Dantas and coworkers at the Baker lab made a breakthrough in protein design. They designed the first de novo 93-residue α/β novel topology protein, which matched the design template with atomic-level accuracy (1.2 Å rmsd) [9]. Such an approach was also implemented in the design of protein–protein and protein–DNA interfaces. In 2004, the Baker lab redesigned the specificity of protein–protein interactions between a nonspecific bacterial DNase (colicinE7) and its inhibitor protein [10]. In 2009, the Baker lab designed the endonuclease I-AniI with a cleavage center on a 20-base-pair DNA target site [11]. In 2013, Baker and colleagues designed a de novo lysozyme inhibitor [12]. In 2014, the Baker lab designed hyper-stable helical bundles, where the resulting designs denatured only at >95 °C and had a 0.4–1.1 Å root-mean-square deviation (RMSD) between the crystal structures and the designs [13]. In 2018, Baker lab designed a fluorescence-activating β-barrel that bound and fluorescently activated DFHBI in vitro and in *Escherichia coli* (*E. coli*), yeast, and mammalian cells [14].

Esterases use a serine-catalyzed triplet to hydrolyze proteins and ester bonds in small molecules. A large number of studies have been carried out on the classical serine/histidine/aspartate (glutamic acid) catalytic triad, where serine is a nucleophile, the imidazole ring of histidine acts as a base. The carboxyl group of aspartic acid (glutamic acid) aligns with the imidazole ring of histidine to neutralize the charge generated on histidine in the transition state [15]. In addition to the catalytic triad, a highly conserved oxyanion binding site (commonly referred to as an oxygen anion hole) stabilizes the negative charge on the carbonyl oxygen of the tetrahedral intermediate [16].

Esterases accelerate the hydrolysis of ester bonds through nucleophilic catalysis. The lone electron pair of the serine hydroxyl oxygen attacks the positively charged carbonyl carbon of the substrate, producing a tetrahedral intermediate stabilized by the catalytic residues, His and Asp/Glu, releasing the alcohol and forming an acyl–enzyme complex. The nucleophile (water in hydrolysis, alcohol or ester in the esterification or transesterification reaction) attacks the acyl–enzyme complex to form a tetrahedral intermediate, which is resolved to give the product (acid or ester) and free enzyme. Esterases usually only accelerate the first step of the ester-cleaving reaction nucleophilic attack on the carbonyl carbon on the substrate ester to give a covalent acyl intermediate [17,18].

If all steps in the catalytic process are designed, multiple processes must be optimized simultaneously, including substrate binding, transition state formation, and product release [19]. To reduce such complexity, this study focused on the most critical step in esterase catalysis—the nucleophilic attack of serine on the substrate’s carbonyl carbon. Serine has an acidity coefficient pKa of ~13, and its acidity is weaker than other amino acids used for nucleophilic catalysis (the pKa of cysteine is about 8). Its nucleophilicity generally depends on the interaction with histidine and aspartate/glutamate in the catalytic triad. Therefore, designing esterases with high nucleophilic serines is challenging, and success is likely to depend on precisely designed hydrogen bonding with the active residues [20].

**Figure 1:**
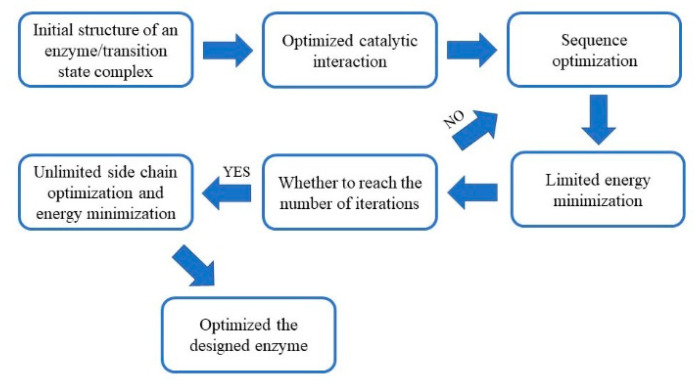
The enzyme design scheme implemented in this study.

Recently, multiple proteins have been designed with unnatural sequences [14,21,22]. However, no research has been performed to design enzymes to meet the demands of industrial applications. In this study, we performed de novo designs and evaluations of an esterase that is widely used as a biocatalyst. The ideal spatial conformation of the catalytic serine triad and the oxygen anion hole bound to the substrate was constructed by quantum mechanical calculations. The catalytic triad and oxygen anion holes were then embedded in the protein scaffold using a de novo designed enzyme protocol in Rosetta 3 (Figure 1). The design results were then evaluated, and the optimal design was used for expression and purification. The designed esterase had significant lytic activities towards p-nitrophenyl acetate (p-NPA).

## 2. Results

### 2.1. Theozyme Optimization by Quantum Mechanics

**Figure 2:**
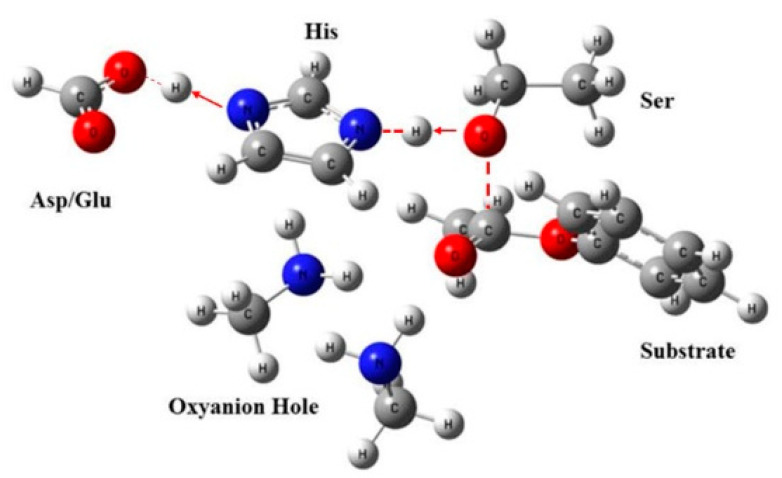
Optimized geometric model of the theozyme. The located transition structure of the theozyme residues is shown as a stick/ball model. Oxygen atoms are in red, nitrogen atoms are in blue, and carbon atoms are in grey.

**Figure 3:**
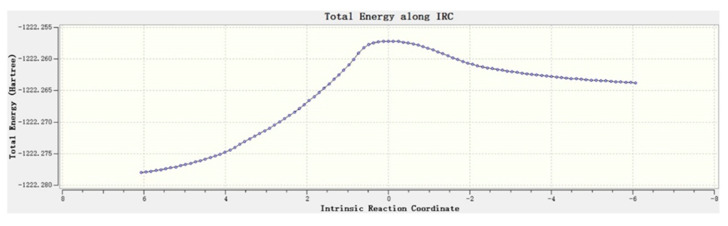
The intrinsic reaction coordinate calculation. The intrinsic reaction coordinate is plotted against the total energy of the system. The energy peaks at the transition state.

The initial theozyme included the side chains instead of entire residues, in which the structures for reactant, transition state, and intermediate were optimized using Gaussian 09 at M06-2X/6-311++G(2d,2p) [23,24] (Figure 2). Vibrational frequency analyses along with intrinsic reaction coordinates calculations showed that such transition states belong to the pathway of nucleophilic attack by the serine residue (Figure 3).

### 2.2. Building of a Protein Skeleton Library

Technically, the complete Protein Data Bank (PDB) can be used as the skeleton. The typical sizes of skeletons range from 100 to 400 residues since proteins smaller than 100 residues are less tolerant to point mutations introduced during the construction of the active site, while the computational cost of proteins larger than 400 residues are too high. In the PDB database, there are 40,066 proteins with 100 to 400 residues, which greatly exceeds the existing computing power. Therefore, in this study, the EzCatDB Database was chosen to build a skeleton library.

The EzCatDB database is based on the PDB database and the enzymes are classified according to their catalytic mechanisms. In addition, the database contains the structural information of various enzymes, which makes it easier to find a suitable skeleton for active site replacement. To reduce the amount of computational work required, the skeleton candidates were limited to those enzymes with any of Ser, His, Asp or Glu at the active site. As a result, 1011 proteins were chosen for further screening.

### 2.3. The Matching between the Skeleton and Theozyme

**Figure 4:**
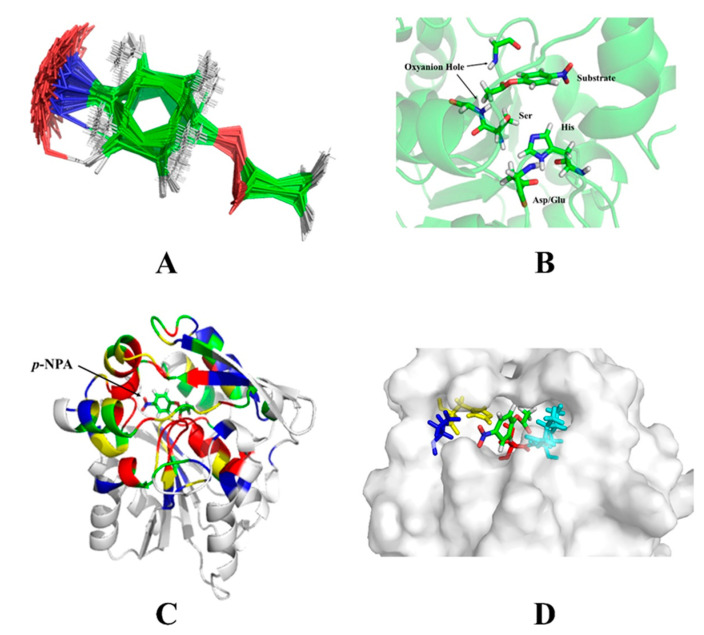
The output of the Rosetta design. (**A**) Superimposition of substrate rotamers. The single bonds in the p-nitrophenyl acetate (p-NPA) substrate were rotated to form various rotational isomers to fit in the active sites of different shapes. The rotating isomers were stacked at the carbonyl carbon to form a set of conformational isomers. (**B**) The initial output structure of RosettaMatch. The catalytic residues, oxyanion and substrate are shown as stick models. The rest of the structure is shown as ribbons. (**C**) The residues in proximity to the substrate. The residues close to the substrate are marked in different colors according to their distances to the substrate. (**D**) The output of RosettaDesign. The substrate is shown in stick models and the designed esterase is shown in the surface model. The substrate fits well in the active site.

The coordinates of the theozyme were converted to a cst file, which is recognizable to the RosettaMatch software. The restraints, such as bond angles, bond distances and dihedral angles, were detailed in the parameter file (Supplement material Doc S1). The substrate conformational isomer set was also converted to a parameter file as an input to the RosettaMatch program (Figure 4A). The RosettaMatch program outputs the grafted model. Since the constraints were relaxed when searching for matching configurations, the grafting model was refined further. Once a match was found, additional amino acids surrounding the active site of the scaffold protein were optimized to construct an active site that conforms to the shape of the substrate and provided a more stable environment for the catalytic residues (Figure 4B).

The residues around the substrate were mutated in different ways according to their distance from the substrate. Amino acid residues with a Cα atom within 6 Å from the heavy atom of the substrate (an atom other than hydrogen) were mutated to any amino acid (the red region in Figure 4C). Residues with a Cα atom within 8 Å of the substrate heavy atom, and the amino acid residue of the Cβ atom pointing to the substrate were mutated to any amino acid (the yellow area). Residues with a Cα atom within 10 Å of the substrate heavy atom were rotationally deformed (the green area). Residues with a Cα atom within 12 Å of the substrate heavy atom and the Cβ atoms pointing to the substrate were rotationally deformed (the blue region). All remaining amino acid residues were unchanged. The interactions between catalytic residues and the substrate were optimized while non-catalytic residues in the active site were mutated to alanine. The freedom of the catalytic residues in the constraint file was reduced, so optimized catalytic conformations were closer to the theozyme’s configurations. Monte Carlo algorithms with the Rosetta standard were used to find lower energy sequences for non-catalytic residues in the active site. The constraints of the catalytic residues were maintained throughout this phase. After the sequence was designed, the resulting structure was subjected to energy minimization for 20 iterations. Then the catalytic constraints were released. The rotamers were optimized for the sequence, and the energy was minimized (Supplement material Doc S2). After Rosetta designed a new sequence, the final rotamer optimization/energy minimization was performed without catalytic residue constraints to check whether the designed sequence maintained the catalytic residue in the catalytic conformation (Figure 4D).

### 2.4. Designed Enzyme Activity Assay

The design results were ranked according to the total score, and the top ten results with the lowest scores were selected as input files for the next round of sequence design. The process was reiterated dozens of times until the sequence did not change in five consecutive designs. The sequences were evaluated and the design results with the lowest total scores were selected for experimental characterization. In total, four protein sequences were selected for characterization. The amino acid sequence and active site conformation are included in the Supplementary material Doc S3.

Based on the wild-type protein gene of the amino acid sequence, the mutation point gene was selected from the *E. coli* preference codon, and the entire gene was ligated into the pET-28a(+) vector and expressed in *E. coli* BL21. The expression results showed that no soluble protein was detected in the supernatant of the four proteins, while inclusion body assays revealed that the 2EBD protein formed inclusion bodies (Figure S1). The remaining three proteins were not effectively detected, indicating that only 2EBD proteins were efficiently expressed (Figure S2). Subsequently, the 2EBD protein inclusion bodies were renatured, concentrated, and purified.

**Figure 5:**
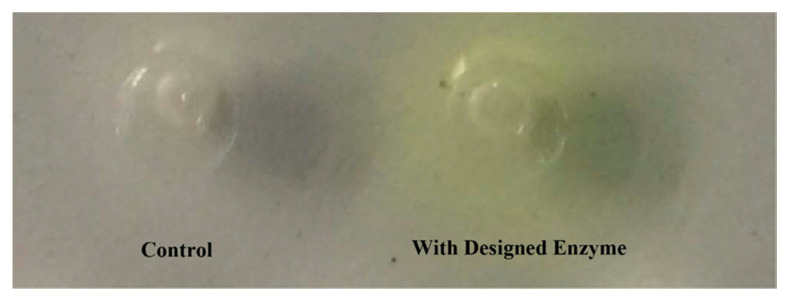
Colorimetric activity assay of the designed esterase. The purified esterase was spotted on an agar plate containing p-NPA. The site containing the enzyme turned yellow, while the site containing a blank solution did not change color.

The activity of the designed enzyme was visually inspected using an agar plate containing p-NPA. The protein was purified via nickel column affinity chromatography and used for the p-NPA hydrolysis assay. The control sample did not hydrolyze p-NPA, and there was no coloration around the pores. Conversely, the pores with the designed enzyme turned yellow, indicating that the designed enzyme had the ability to hydrolyze p-NPA (Figure 5).

Next, the catalytic enzyme activity of the designed enzyme was quantitatively determined. The protein concentration of the enzyme was 2.841 ± 0.014 mg/mL. Enzyme activity assays were carried out at 37 °C for 5 min, revealing that the activity of 50 μL of enzyme solution was 31.035 ± 0.107 U/g protein.

To confirm that the activity of the designed enzyme was attributed to the designed active site, residues in the catalytic triad and the oxyanion hole were sequentially mutated to alanine to determine their contributions to the enzyme activity. The S307A, H174A, and E118A mutants of the catalytic triads, as well as the K308A and F309A mutants of the oxygen anion holes formed inclusion bodies in *E. coli* (Figure S3). The inclusion bodies of the five mutants were renatured, and the refolded proteins were purified by nickel affinity chromatography (Figure S4). The purified mutant proteins were assayed for enzymatic activity with p-NPA under the same conditions as the designed enzyme. The mutation of any one of the above five sites to alanine greatly decreased the catalytic activity (Table 1).

In addition, the substrate template protein, 2EBD, was also studied. The 2EBD protein is a 3-oxoacyl-[acyl-carrier-protein] synthase III from the hyperthermophilic *Aquifex aeolicus* (Uniprot ID O67185). That protein catalyzes the first condensation reaction that initiates fatty acid synthesis, and thus, may play a role in controlling the overall rate of fatty acid production. The protein has the activity of an acetyl–acyl carrier protein synthetase and an acetyltransacylase. Its substrate specificity determines the biosynthesis of branched and/or linear fatty acids. The catalytic activity sites are Cys111, His236, Asn266.

The catalytic triad designed in this study included Ser307, His174, and Glu118 in the designed protein, which corresponded to Leu307, Ile174, Leu118 in the template that are not the catalytic residues. The template protein itself did not have the ability to hydrolyze p-NPA, and therefore, the p-NPA hydrolyzing activity of the designed protein’s active site was designed from scratch.

## 3. Discussion

In this work, we designed a de novo esterase by constructing catalytic triads and oxygen anion holes, and matching them with suitable skeletons. Subsequent activity assays indicated that the designed esterase had activity towards p-NPA. The commercial esterase available at Sigma-Aldrich has an activity of 10^4^–10^5^ U/g, which is about 1000 times the activity of the designed enzyme in this study. It has been shown that most computational-designed enzymes are much less active than the natural enzymes [25]. Although the esterase that we designed in this work is not as good as the commercial enzymes, it still paves the way for further optimization, which may lead to a highly-efficient enzyme with good commercial values. The common set of catalytic residues in hydrolases, including Ser-His-Asp/Glu, was chosen as the catalytic triad, and two NH groups in the adjacent backbone were selected to form the oxygen anion hole. Other studies used Cys-His catalytic dyads as catalytic residues, in which the catalytic activity was attributed to Cys. The active site cysteines were rapidly acylated in the reaction, but the slow hydrolysis of the acyl–enzyme intermediate led to a low overall catalytic efficiency [17].

The hydrogen bonding strength between histidine and serine is more favorable for proper positioning than histidine and cysteine. The nucleophilicity of serine usually depends on the catalytic triad with activating residues such as histidine and aspartic acid or glutamic acid, so the stability of the catalytic triad is also an important factor in increasing the catalytic activity [20]. The structural parameters of the catalytic triad are also more accurate. Oxygen anion holes composed of two NH groups are also important in the catalytic process. The oxyanion holes formed by backbone NH groups, side-chain NH groups or water molecules were compared to each other. It was found that all active design enzymes use a backbone NH group to stabilize the oxyanion, while the use of a side chain NH group or water molecule to stabilize the oxyanion was inactive [17].

In the present study, the main chain NH group was used to stabilize the oxyanions formed on the substrate carbonyl oxygen (Figure S5). The backbone oxygen anion hole is found in almost all proteases and many esterases. In general, the protein backbone is more rigid than the side chain [26,27,28,29]. Therefore, when screening the designed results, it may be preferable to choose a design that uses a backbone NH group to form an oxygen anion hole. In this study, any one of the residues in the mutant oxyanion hole reduced the catalytic activity, but the decrease was not as significant as that caused by mutation at the triad. This may be due to the fact that the mutated alanine also has a backbone NH group that provides the stabilizing force to the oxyanion.

“Theozymes” refers to an enzyme model that theoretically stabilizes the transition state of a reaction, in which quantum mechanical methods are used to predict the spatial position of catalytic residues [30,31]. The spatial configuration of the theozymes (bond length, bond angle, dihedral angle) was calculated by quantum mechanics. It is particularly important to use a more appropriate method and basis set to interpret the interactions between the residues at the active site. The M06-2X method and the 6-311++g(2d, 2p) basis set used in this experiment had a theoretical level of accuracy, convergence, and computational speed at the current computational power. With the development of computing power and the improvement of computational methods for quantum chemistry theory, better and faster calculation methods will be developed to construct transition states. In addition, the esterase hydrolysis process is a multi-step conversion process. In this study, only the theozymes that catalyze the acylation step were designed, which may reflect the fact that the transition state constructed in this experiment cannot accelerate the complete process of the multi-step transformation. Conversely, natural enzymes may accelerate the multiple steps of the entire catalytic process. The solution to this problem requires superimposing the theozymes that stabilize the transition state of multiple steps to search for the active site structure with the smallest shape change. As a theozyme that catalyzes the entire process, this process requires substantial computing power, which may be accomplished with large computer clusters.

The catalytic triad in the synthesized enzyme was buried deeper in the active site cavity, ensuring the proper interaction with the surrounding amino acids. Moreover, the hydrophobic active site and the position of active residues deep in the active site avoid external solvents, making it easier to maintain the structure conformation. The design process made major changes to the amino acid sequence of the scaffold protein and constructed a cavity structure that was different from the original one, which may have had a significant impact on protein folding. Among the four enzymes selected, one enzyme formed inclusion bodies, while the other three were not expressed at detectable levels. In general, most in silico designed enzymes are unable to be expressed well or are insoluble. Low expression levels are another factor that affects the success rate of enzyme designs.

On average, each de novo designed enzyme introduces 15 mutations into its natural protein scaffold, and each mutation adds to the instability of the protein. Thus, the cumulative effects of multiple mutations can easily destroy the overall integrity of the protein [32]. Several methods can be applied to overcome this obstacle. For example, thermophilic proteins are more tightly packed and more tolerant to mutations, which can serve as a good starting point for introducing large numbers of mutations. Additionally, through rational design, residues in regions not related to catalytic function may be altered to increase the solubility of the enzyme. In some cases, a small number of mutations is sufficient to increase the melting temperature by 10° C without reducing catalytic efficiency [33].

## 4. Materials and Methods

### 4.1. Materials

The computational designs were run on an Intel^®^ Xeon^®^ Processor X5690 processor (12M Cache, 3.46 GHz, 6.40 GT/s Intel^®^ QPI) using the CentOS 6.4 operating system. We would like to thank our collaborators at Jilin University and Huazhong Normal University for providing the resources needed for quantum chemical calculations. The Rosetta package was downloaded from http://www.rosettacommons.org.

Restriction endonuclease, Taq DNA polymerase, DNA ligation kits and PrimeSTAR^®^ HS DNA polymerase were purchased from TaKaRa (Otsu, Japan). Kits for plasmid and gel extractions were purchased from Omega Bio-tek (Norcross, GA, USA). p-Nitrophenyl (p-NP) ester was purchased from Sigma-Aldrich (St. Louis, MO, USA). Reduced glutathione and oxidized glutathione were purchased from Merck (Darmstadt, Germany). Protein marker was purchased from ThermoFisher Scientific (Waltham, MA, USA). All other chemicals were purchased from China Pharmaceutical Chemical Reagent Co., Ltd. (Shanghai, China).

### 4.2. The Construction of Theozymes

The transition state of the first step of the catalytic reaction (i.e., the substrate ester subject to a nucleophilic attack by serine) was investigated. The theozyme consisted of a Ser-His-Glu/Asp triad and an oxygen anion hole composed of two residue side chains. The reactants, transition states, and products were optimized using Gaussian 09 at the M06-2X/6-311++g(2d, 2p) level. Each state was subjected to vibrational frequency analyses to confirm the local minimum or the transition state. In addition, the intrinsic reaction coordinates (IRC) calculations were performed to verify the accuracy of the transition state.

### 4.3. Matching between Theozymes and Protein Skeleton Library

In order to reduce the number of calculations, the EzCatDB database was used to search for the enzyme structure containing Ser, His, Glu or Asp in the active site, and the result was used as a skeleton structure library for the theozyme [34,35]. Matching between theozymes and the protein skeleton library was performed in two steps using RosettaMatch [36]. Firstly, each catalytic side chain in the theozyme was attached to the backbone within the active site region of the scaffold protein. Secondly, RosettaMatch was used to analyze the positions of the side chains and theozymes to identify the spatial conflicts with the scaffold protein.

RosettaDesign was then used to redesign the area near the active site to stabilize the enzyme geometry without significantly affecting protein stability [37]. When RosettaMatch generated a combination of scaffold proteins and theozymes, the RosettaDesign program was used to introduce mutations into the non-catalytic residues at the active site, making the active site conformation more compatible with the transition state. In addition, some of the free residue side chains were rotated to optimize the configuration.

The process first involved iteratively optimizing the position of the transition state so that the various parameters of the catalysts were as close as possible to the ideal state. The three-dimensional structures of those sequences was then iteratively optimized while maintaining the integrity of the catalytic system [38]. When the maximum number of iterations was reached, the restriction of the catalytic residue was removed and the entire system was subjected to rotational isomer optimization to achieve the lowest energy. Subsequently, the structure of the RosettaDesign output was sorted.

### 4.4. The Evaluation Screening and Sorting of the Designed Enzyme

RosettaDesign outputs hundreds of model structures. However, only one or several were selected for subsequent experimental characterizations, which required evaluation and screening of the putative enzyme models. This study adopted a widely-used screening method. The total score should not be greater than the total score of the original scaffold protein. The ligand binding energy was less than −10.0 Rosetta energy units (REU) (SR_interface_1_2 < −10.0), the total score of the catalytic residue conforming to the constraint range was less than 1 (all_cst < 1.0), the number of the polar atoms that were unsaturated and buried in the ligand and catalytic residues was no more than two (SR_burunsat_pm ≤ 2), and more than 66% of the ligands’ surface was buried in the protein (SR_dsasa_1_2 > 0.66) [39,40]. The designed enzyme results were sorted according to the total score, and the best-designed results were experimentally characterized.

### 4.5. Gene Synthesis of the Designed Enzyme, In Vitro Expression, and Purification

Based on the wild-type gene of the protein skeleton, the corresponding mutation was introduced through whole gene synthesis. The gene for the synthetically designed enzyme was cloned into pET-28a(+) and expressed in *E. coli* BL21 cells. The protein expression was induced by 0.1 mM isopropyl β-d-1 thiogalactopyranoside (IPTG), and the cells were harvested after incubation by centrifugation at 4000× *g* for 15 min. The cell pellet was resuspended in 20–30 mL of pre-cooled NTA-0 buffer (0.05 M Tris-HCl, 0.5 M NaCl, pH 8.0), and lysed through sonication. The supernatant and the precipitate were collected by centrifugation at 12,000 rpm for 30 min at 4 °C. A small amount of supernatant and precipitate was used for SDS-PAGE detection and the remainder was stored at 4 °C until use. The pellet was resuspended in 50 mL NTA-0 buffer and dithiothreitol (DTT) was added to a final concentration of 1 mM. Ultrasound was used to promote the dissolution of the heteroprotein and the supernatant was removed by centrifugation at 12,000 rpm for 10 min at 4 °C. The above three steps were repeated until the supernatant was clear. The pellet was then resuspended in PBS and ultrasound was used to promote dissolution. Samples were then centrifuged at 12,000 rpm for 10 min at 4 °C to remove the supernatant. Inclusion bodies were then resuspended in 3 mL of 6 M guanidine hydrochloride and DTT was added to a final concentration of 5 mM. The mixture was shaken at 200 rpm for 3 h at 37 °C until the inclusion bodies were completely dissolved. The mixture was then centrifuged at 12,000 rpm for 10 min at 4 °C, and the supernatant was subjected to SDS-PAGE detection.

The protein solution was diluted with 3 M guanidine hydrochloride and added to a 200 mL reconstituted solution (pH 8.0) with a syringe at 4 °C. The resulting solution was stirred at maximum speed for 24 h. The resulting protein solution was concentrated to 50–100 mL using PEG 20,000 and the concentrated solution was dialyzed overnight in PBS buffer at 4 °C. The volume was then concentrated to 2–4 mL with PEG 20000. After subsequent overnight dialysis at 4 °C, the protein solution was subjected to SDS-PAGE detection. The protein supernatant was filtered using a 0.22 μm filter and added to the Ni-NTA column. The column was washed with 30 mL NTA-0 buffer to remove impurities and gradient elution was performed using NTA-0 buffer containing different concentrations of imidazole (0.03 M, 0.06 M and 0.5 M). The eluate was subjected to SDS-PAGE detection.

### 4.6. Activity Assay of the Designed Enzyme

A total amount of 1.5–2 g of agar powder was added in 100 mL of ultrapure water and melted by heating. After cooling to 50–60 °C, 200 μL (100 mM) of p-NPA was added, followed by preparation of a plate. A puncher was used to generate a hole with 4–5 mm diameter in the agar plate and the purified enzyme solution was added to the well. The plate was incubated at 37 °C to observe changes in the hydrolysis zone. A total of 1 mL of the reaction system contained 10 μL of p-NPA (100 mM), 40 μL of ethanol, 900 μL of Tris-HCl buffer (50 mM, pH 8.0) and 50 μL of enzyme solution. In the blank control, the same volume of Tris-HCl buffer was used instead of the enzyme solution. The activity was determined with the colorimetric assay by measuring the absorbance at 410 nm. Additionally, the degradation was visually inspected by the observation of yellow coloration. The experiment was carried out at 37 °C for 5 min. One unit (U) of esterase activity was defined as the amount of enzyme that released 1 μmol of p-NP per minute under the assay conditions described. To verify that the catalytic activity was indeed derived from the designed catalytic active site, alanine mutations were introduced to the active site residues. The enzyme activities before and after the mutation were compared to confirm the activity of the designed enzyme.

## Figures and Tables

**Table 1:** Catalytic activity (U/g) determination after alanine mutation of catalytic residues.

Designed Enzyme	S307A	H174A	E118A	K308A	F309A
31.035 ± 0.107	N.A.	N.A.	1.001 ± 0.016	8.914 ± 1.497	5.402 ± 2.050

## Supplementary Materials

The following are available online, Figure S1: SDS-PAGE of designed 2EBD protein, Figure S2: SDS-PAGE of inclusion bodies of four designed enzymes, Figure S3: SDS-PAGE of 2EBD mutants, Figure S4: SDS-PAGE of 2EBD mutants after refolding and purification. Figure S5: Analysis of oxyanion hole in designed enzymes.

## Abbreviations

p-NPA	p-Nitrophenyl Acetate
