# Supplementary material for: A De Novo Designed Esterase with p-Nitrophenyl Acetate Hydrolysis Activity
Source: Molecules. 2020 Oct 13;25(20):4658. doi: 10.3390/molecules25204658 (PMC7587395; doi:10.3390/molecules25204658)
Supplement: Supplementary file 1 [file molecules-25-04658-s001.pdf]

Supplementary Material

A De Novo Designed Esterase with p-Nitrophenyl Acetate Hydrolysis Activity

Guanlin Li[1], Houjin Zhang[1], Junjun Liu[2], Li Xu[1], Jinyong Yan[1], Yunjun Yan[1,*]

1. Key Laboratory of Molecular Biophysics, Ministry of Education, College of Life Science and Technology, Huazhong University of Science and Technology, Wuhan, People's Republic of China
2. School of Pharmacy, Tongji Medical College, Huazhong University of Science and Technology, 13 Hangkong Road, Wuhan, People's Republic of China

* Correspondence: yanyunjun@hust.edu.cn

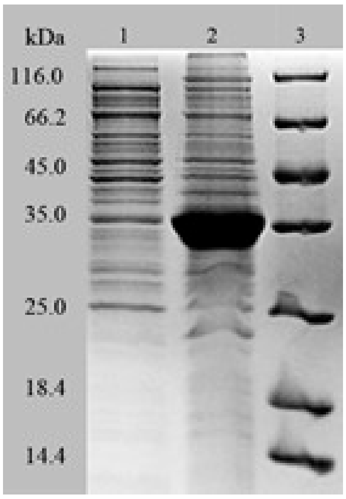

Figure S1. SDS-PAGE of designed 2EBD protein. Lane 1, supernatant; Lane 2, inclusion bodies; Lane 3, protein marker.

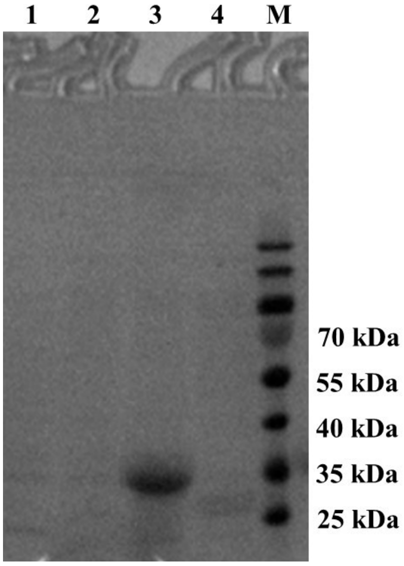

Figure S2. SDS-PAGE of inclusion bodies of four designed enzymes. Lane 1, 1CUW-based enzyme; Lane 2, 2YAS-based enzyme; Lane 3, 2EBD-based enzyme; Lane 4, 3GBS-based enzyme; Lane M, Marker.

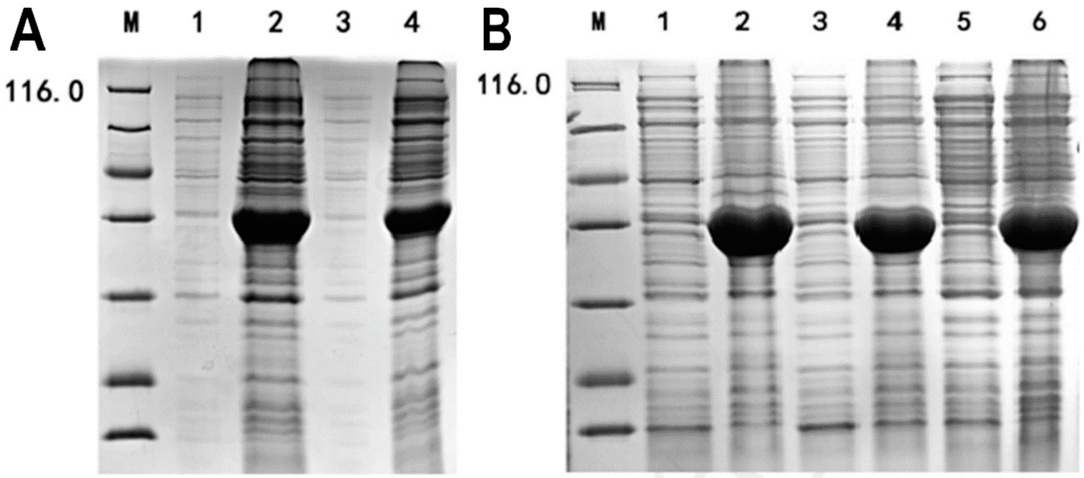

Figure S3. SDS-PAGE of 2EBD mutants. Lane M: Protein molecular weight standards (116.0/66.2/45.0 /35.0/25.0/18.4/14.4 kDa). (A) Lane 1/2: Supernatant and pellet of E118A; Lane 3/4: Supernatant and pellet of H174A; (B) Lane 1/2: Supernatant andpellet of S307A; Lane 3/4: Supernatant and pellet of K308A; Lane 5/6 : Supernatant and pellet of F309A.

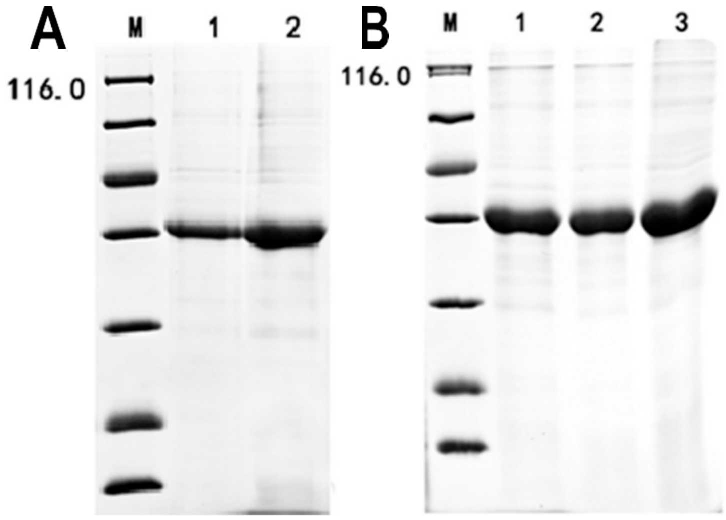

Figure S4. SDS-PAGE of 2EBD mutants after refolding and purification. Lane M: Protein molecular weight standards (116.0/66.2/45.0/35.0/25.0/18.4/14.4 kDa). (**A**) Lane 1:E118A; Lane 2: H174A; (**B**) Lane 1: S307A; Lane 2: K308A; Lane 3: F309A**.**

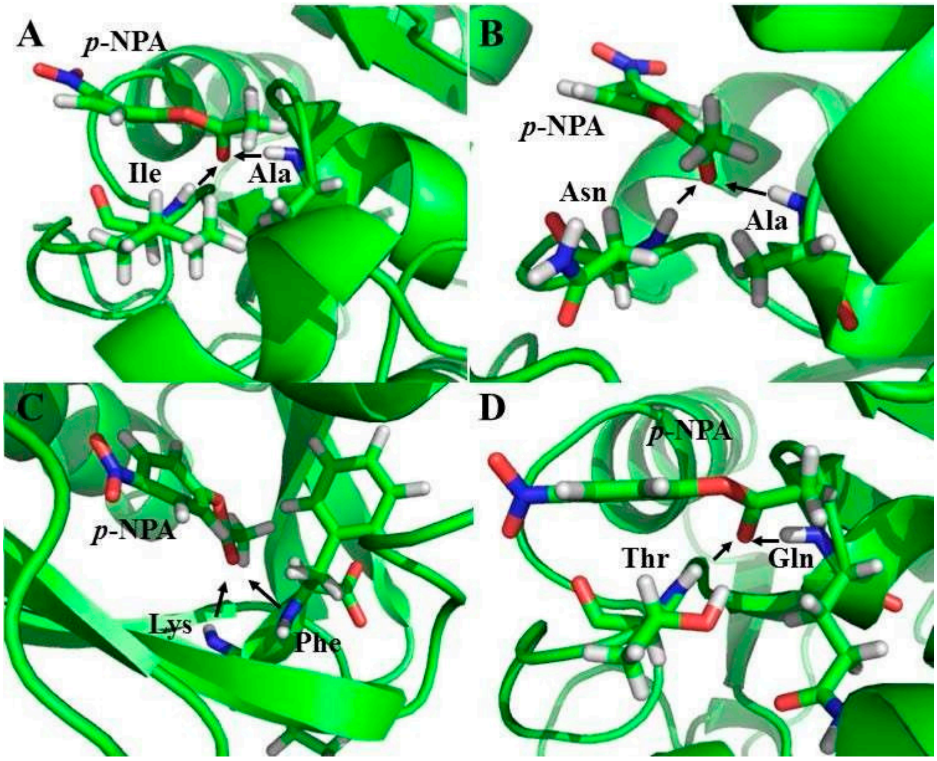

Figure S5. Analysis of oxyanion hole in designed enzymes. (A) 1CUW-based esterase; (B) 2YAS-based esterase; (C) 2EBD-based esterase; (D) 3GBS-based esterase.

Doc S1 Restrain file for RosettaMatch.

#block 1
Substrate_Ser_interaction
CST::BEGIN
TEMPLATE:: ATOM_MAP: 1 atom_name: C7 O3
C6 TEMPLATE:: ATOM_MAP: 1 residue3: PNP
TEMPLATE:: ATOM_MAP: 2 atom_type: OH
TEMPLATE:: ATOM_MAP: 2 residue1: S
CONSTRAINT:: distanceAB: 1.88 1.00 180.00 1
5
CONSTRAINT:: angle_A: 97.72 15.00 100.00 360.00 3
CONSTRAINT:: angle_B: 114.81 15.00 100.00 360.00 3
CONSTRAINT:: torsion_A: 83.10 20.00 50.00 360.00 2
CONSTRAINT:: torsion_AB: -112.09 60.00 50.00 360.00 2
CONSTRAINT:: torsion_B: -122.49 40.00 50.00 360.00
2 ALGORITHM_INFO:: match
IGNORE_UPSTREAM_PROTON_CHI
ALGORITHM_INFO::END
CST::END
#block 2
Ser_His_interaction
CST::BEGIN
TEMPLATE:: ATOM_MAP: 1 atom_type:
OH TEMPLATE:: ATOM_MAP: 1
residue1: S

TEMPLATE:: ATOM_MAP: 2 atom_type:
Nhis TEMPLATE:: ATOM_MAP: 2 residue1:
H CONSTRAINT:: distanceAB: 2.51 0.60
100.00 0 3
CONSTRAINT:: angle_A: 109.47 15.00 30.00 360.00 3
CONSTRAINT:: angle_B: 125.21 20.00 30.00 360.00 4
CONSTRAINT:: torsion_AB: -42.81 20.00 30.00 60.00 2
CONSTRAINT:: torsion_B: -179.17 40.00 30.00 360.00
2 ALGORITHM_INFO:: match
IGNORE_UPSTREAM_PROTON_CHI
SECONDARY_MATCH: UPSTREAM_CST 1
ALGORITHM_INFO::END
CST::END
#block 3
His_Asp_interaction
CST::BEGIN
TEMPLATE:: ATOM_MAP: 1 atom_type:
Ntrp TEMPLATE:: ATOM_MAP: 1
residue1: H
TEMPLATE:: ATOM_MAP: 2 atom_type: OOC
TEMPLATE:: ATOM_MAP: 2 residue1: D
CONSTRAINT:: distanceAB: 2.55 0.40 100.00 0 2
CONSTRAINT:: angle_A: 132.44 15.00 30.00 360.00 3
CONSTRAINT:: angle_B: 111.16 60.00 30.00 360.00 3
CONSTRAINT:: torsion_A: -179.79 30.00 30.00 360.00 2
CONSTRAINT:: torsion_AB: 179.33 20.00 30.00 90.00 2
CONSTRAINT:: torsion_B: -180.00 20.00 30.00 90.00
2 ALGORITHM_INFO:: match
IGNORE_UPSTREAM_PROTON_CHI
SECONDARY_MATCH: UPSTREAM_CST 2
ALGORITHM_INFO::END
CST::END

#block 4
oxyanion_hole_1_bb
CST::BEGIN
TEMPLATE:: ATOM_MAP: 1 atom_name: O4 C7
O3 TEMPLATE:: ATOM_MAP: 1 residue3: PNP
TEMPLATE:: ATOM_MAP: 2 atom_type:
Nbb TEMPLATE:: ATOM_MAP: 2
is_backbone
TEMPLATE:: ATOM_MAP: 2 residue1:
ACDEFGHIKLMNQRTVWY CONSTRAINT:: distanceAB: 3.07 0.30
20.00 0 2
CONSTRAINT:: angle_A: 145.45 60.00 20.00 360.00 2
CONSTRAINT:: angle_B: 114.42 20.00 20.00 360.00 2
CONSTRAINT:: torsion_A: 155.69 30.00 20.00 360.00 3
CONSTRAINT:: torsion_AB: -70.47 20.00 20.00 360.00
2 ALGORITHM_INFO:: match
SECONDARY_MATCH:
DOWNSTREAM
ALGORITHM_INFO::END

CST::END
#block 5
oxyanion_hole_2_bb
CST::BEGIN
TEMPLATE:: ATOM_MAP: 1 atom_name: O4 C7
O3 TEMPLATE:: ATOM_MAP: 1 residue3: PNP
TEMPLATE:: ATOM_MAP: 2 atom_type:
Nbb TEMPLATE:: ATOM_MAP: 2
is_backbone
TEMPLATE:: ATOM_MAP: 2 residue1:
ACDEFGHIKLMNQRTVWY CONSTRAINT:: distanceAB: 2.97
0.30 20.00 0 2
CONSTRAINT:: angle_A: 145.10 10.00 20.00 360.00 2
CONSTRAINT:: angle_B: 103.11 20.00 20.00 360.00 2
CONSTRAINT:: torsion_A: -61.80 20.00 20.00 360.00 2
CONSTRAINT:: torsion_AB: 54.08 20.00 20.00 360.00
2 ALGORITHM_INFO:: match
SECONDARY_MATCH:
DOWNSTREAM
ALGORITHM_INFO::END
CST::END

Doc S2 The script for matching optimization

-enzdes

-cstfile inputs/enzdes_asp_his_ser.cst // At this time, the parameter file with more restrictions is used.

-detect_design_interface // Automatic detection of substrate position

-cut1 6.0

-cut2 8.0

-cut3 10.0

-cut4 12.0

-fix_catalytic_aa // Fixed catalytic residues without mutation or rotation

-enzdes

-cst_opt //Calling the optimization process

-bb_min //Allowing the scaffold protein to move slightly when minimized

-chi_min //Allowing dihedral angles of catalytic residues to change during minimization

-enzdes

-cst_design //Calling the design process

-design_min_cycles 20 //Design/minimized iterations

-lig_packer_weight 1.6 //Determine protein/substrate interactions and proportion of protein/protein interactions in sequence selection calculations

-cst_min //Calling the energy minimization process after sequence design

-bb_min //Same as the second step

-chi_min //Same as the second step

-packing //Calling the rotation isomer optimization process

-ex1 //Improve rotatomer sampling around the first dihedral angle of each amino acid

-ex2 //Improve rotatomer sampling around the second dihedral angle of each amino acid

-use_input_sc //Use the side chain rotamer in the input file for calculations

-soft_rep_design //Use a soft repulsive force field in the design

-linmem_ig 10 //Speed up the sequence design while reducing memory requirements

-packing //Calling the rotation isomer optimization process

-ex1 //Same as the third step

-ex2 //Same as the third step

-use_input_sc //Same as the third step

-enzdes //Call energy minimization process

-cst_min //Same as the third step

-bb_min //Same as the third step

-chi_min // Same as the third step

Doc S3 Designed enzyme sequences

Designed enzyme No.1 (use 1CUW as the scaffold protein, designated as 1CUW enzyme)

MAPTSNPAQELEARQLGRTTRDDLINGNSASCADVIFIYARGIGETGNLGKLGP
SIASNLESAFGKDGVWIQGVGGAYRGTEAERFLPRGTSSAAIREMLGLFQQAN
TKCPDATLIAGGRSAGAALAAASIEDLDSAIRDKIAGTVLFGYIKNLQNRGRIP
NYPADRTKVFCNTGDLTCTGSRIKLAPHDAYGPDARGPAPEFLIEKVRAVRGS A

Designed enzyme No.2 (use 2YAS as the scaffold protein, designated as 2YAS enzyme)

MAFAHFVLIPGNGGGAWQWHKLKPLLEALGHKVTALDLAASGVDPRQIEEIG
SFDEYSEPLLTFLEALPPGEKVILVGHSAGGLNIAIAADKYCEKIAAAVFHNSLL
PDTEHCPSYVLDKLMEVFPDWKDTTYFTYTKDGKEITGLKYGFTLLRENFHT
LCGPEEYELAKMGQRKGSLFQNILAKRPFFTKEGYGSIKKIYVWTDQDEYDL
PEFQLWQIENYKPDKVYKVEGGDHNLQLTKTKEIAEILQEVADTYN

Designed enzyme No.3 (use 3GBS as the scaffold protein, designated as 3GBS enzyme)

MHLRNIVIALAATAVASPVDLQDRQLTGGDELRDGPCKPITFIFARSTGEPGLL
GISTGPAVCNRLKLARSGDVACQGVGPRYTGDFPSLALPEGTSQAAIAEAQGL
FEQAVSKCPDTQIVAGGQSQGTAVMNGAIKRLSADVQDKIKGVVLFGYLRNA
QERGQIANFPKDKVKVYCAVGDLFCLGTLIKAPPHDSYLSDTGDASDFLLSQL G

Designed enzyme No.4 (use 2EBD as the scaffold protein, designated as 2EBD enzyme)

MGTTTIGTGVYLPKNVLTNFDLEKIVDTSDEWITTRTGIKERRIAKEETITYMA
TQAAKEALREANLSPEELDLIILATLTPQKRFPSTACLVQAQLKAKGVYAFDIS
AACSGFLYA***E***DIADSFIKSGKAKNVLVIGAEKLSEAVDWEDRSTCVLFGDGAG
ATVQTRSEDKSD***H***LATRMYAEGSLEELLHADNCGYIRMKGRELFKVAVRSME
EVCREVLEKAGVKPEEVSWVNPHQANVRIINALAEKLNIPKEKVFVNIQKYG
NTSAASLPIALHERIKEGKLKRGDLSLATAMGGGLTWGAML***SKF***

Table S1. Frequency calculation of the transition state.

Label	Frequencies	Infrared
1	−274.68	590.5490
2	14.73	0.1057
3	18.52	0.2515
4	21.27	0.2790
5	24.80	0.0868
6	36.97	0.5024
7	45.61	1.1116
8	49.60	0.3349
9	58.30	0.6460
10	68.34	0.2352
